# Application of the Rasch measurement model in rehabilitation research and practice: early developments, current practice, and future challenges

**DOI:** 10.3389/fresc.2023.1208670

**Published:** 2023-07-17

**Authors:** Alan Tennant, Ayse A. Küçükdeveci

**Affiliations:** ^1^ Leeds Institute of Rheumatic and Musculoskeletal Medicine, University of Leeds, Leeds, United Kingdom; ^2^ Department of Physical Medicine and Rehabilitation, Faculty of Medicine, Ankara University, Ankara, Turkey

**Keywords:** Rasch, rehabilitation, guidance, history, stroke

## Abstract

The application of the Rasch measurement model in rehabilitation is now well established. Both its dichotomous and polytomous forms provide for transforming ordinal scales into interval-level measures, consistent with the requirements of fundamental measurement. The growth of applying the model in rehabilitation spans 30 years, during which both the protocol has steadily developed and several software packages have emerged that provide for analysis, together with the “R” language that has an increasing set of codes for applying the model. This article reviews that development and highlights current practice requirements, including those for providing the relevant information for the methods, and what is expected of the analysis. In addition, this provides a worked example and looks at the remaining issues and current developments of its application.

## 1. Introduction

It has been over 30 years since Rasch analysis was introduced to rehabilitation, based on the original work by Georg Rasch, a Danish mathematician and statistician. Its application in rehabilitation to date has been prolific, with almost 1,500 manuscripts indexed in PUBMED with “Rasch” as a title or abstract together with “rehabilitation”. These range from early works examining existing scales, through the development of new scales, to the development of item banks for computer adaptive testing (CAT) (1–3). Why then should it be so popular in the context of assessment and measuring outcomes in rehabilitation? As such, it seems appropriate to review what exactly the model is, how it came to be applied in rehabilitation, and what the current practice and issues that arise are, together with possible future developments.

### 1.1. What is the Rasch model?

**Figure 1:**
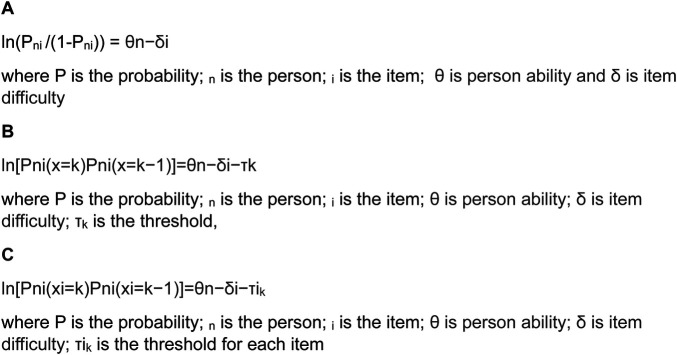
The Rasch model parameterisations in log-odds format. (**A**) The dichotomous model. (**B**) The rating scale model. (**C**) The partial credit model.

In its simplest (dichotomous) form, it is a probabilistic model that postulates that the probability of obtaining a correct response to a test item (e.g. correct/incorrect) is a logistic function of the difference between the person's ability and the difficulty of the item presented (4). In its log-odds format, it is simply the difference between the person’s ability and item difficulty (Figure 1A). Essentially, much of rehabilitation assessment is about contrasting the ability of the person undergoing rehabilitation against a series of tasks of often increasing difficulty. In this way, “by applying the Rasch model, formulating (clinical) expectations about what should happen when a group of persons take a test can be made, and compared to the empirical findings produced by the responses to the scale for the person under investigation” (5).

It was almost two decades after Rasch produced his dichotomous model that it was extended to cover what we now consider as rating scales, for example, a five-point response option for each item, representing an increase in either ability or disability. This is known as the polytomous form of the model, and it consists of two versions known as the rating scale and partial credit formulations (6, 7). Essentially, in these forms of the model, the probability is in the transition from one category to the next, that transition point being known as the threshold (τ). However, in the early literature, it was also known as the “step” (8). The difference between the two forms of the polytomous model is that the rating scale model required the distances between thresholds to be the same across all items (Figure 1B), while the partial credit item allowed these distances to vary [τi] (Figure 1C). While in fact most assessments are structured in such a way that the rating scale model would seem the obvious choice, in practice empirically, this is rarely the case as distances between thresholds vary across items, irrespective of how the categories are presented to patients (9). It was this polytomous format of the model that drove the rehabilitation interest mainly as many of the assessments were of this nature, perhaps the most famous of which, at that time, was the Functional Independence Measure (FIM™) (10). The development of the polytomous model also coincided with two influential developments for model application. The first influence was the emergence of computer software to implement the model, notably the BICAL, BIGSTEPS, WINSTEPS, and later the RUMM programmes (11–14). The second influence was the MESA Psychometric Laboratory, at The University of Chicago, and its teaching manuals “best test design” and “rating scale analysis” (15, 16). It is no coincidence that early applications of the Rasch model in rehabilitation were associated with this laboratory where Ben Wright and Mike Linacre would run introductory workshops attended by many of the early practitioners of Rasch analysis in rehabilitation (8, 10, 17–20).

### 1.2. Why is the Rasch model special?

To understand why the Rasch model is so special, it is necessary to go back almost 60 years to the seminal work by Luce and Tukey (21). Their work, “Simultaneous conjoint measurement: A new type of fundamental measurement” laid the foundation for converting ordinal scales into interval-level measurement, given certain requirements were met. Ordinal scales allow the measurement of the magnitude of a trait, but where the distances between raw score points are not equal, and so mathematical calculations such as change scores or standard deviations are inadmissible (22, 23). FIM™ and most other non–instrument-based assessments used in rehabilitation, including all summative patient-reported outcome measures (PROMs), are ordinal scales, summing the responses to items to give a total (or subscale) score. Hence, there is a need to find a way to convert ordinal scales to interval-level measurement if, for example, change scores are required to monitor rehabilitation progress. It must be stated that, with ordinal scales, changes in magnitude can also be recorded, but without the ability to infer by how much change.

As such, the Rasch model has been reported as a special case of additive conjoint measurement where the fit of data to the model implies that items and persons are measured on an interval scale with a common unit (24). Thus, fitting data from an assessment to the Rasch model allows clinicians and researchers to use a respondent's raw test or scale score and to express the respondent's performance on a linear scale that accounts for the unequal difficulties across all test items (25). The ability of the Rasch model to deliver this transformation was widely promoted in the early literature, but it was not until 2009 that the mathematical proof of the Rasch model as a probabilistic form of conjoint measurement was published (26–28).

### 1.3. What attributes of the Rasch model make it a special case of additive conjoint measurement?

The key to understanding its role in conjoint measurement is in understanding the implications of Rasch's term specific objectivity, which means that the model permits the comparison of two subjects independent of which stimuli are used to measure them, as well as the comparison of two stimuli independent of the subjects on whom they are used (29). This means that the measurement of a person’s ability is independent of the distribution of items in the scale, and also the calibration of item difficulty is independent of the distribution of the person’s ability in the sample. Also, out of all the models within the general item response theory (IRT) framework, the Rasch model is unique because it is the only parametric model where the raw score over all items is a sufficient statistic for the person parameter. Due to this property, conditional maximum likelihood (CML) estimation can be used to estimate item parameters consistently without assuming a specific population distribution for the latent trait. For example, the relative difficulty/impact of any two items can simply be derived from their comparative responses given (conditioned on) the total score.

Furthermore, data fitting the model satisfies the requirement of homogeneity and is the only parametric model to do so. This means that the ordering (difficulty) of items remains the same, irrespective of where a person is on the trait being measured (i.e. their ability level). It is also not possible for a person to answer different items with varying levels of ability (30).

## 2. What are the requirements for data to fit the Rasch model?

A set of requirements need to be satisfied if a set of data is consistent with the Rasch model and thus to attain an interval-level transformation (Table 1) (31). Most modern software such as RUMM2030, WINSTEPS, or DIGRAM and, increasingly, R-based code, provide for testing these requirements (13, 14, 32–35). The former two require purchase, DIGRAM is giftware, and R-based code for Rasch is also free.

**Table 1:** Requirements of the Rasch model.

Requirement	Definition	Way of testing
Homogeneity	The same hierarchical ordering of items should hold for each level (or grouping) of the score	Item/person fit by groups of the raw score. Chi-square; Infit/outfit
Local item (response) Independence	Correlations between items should be zero after conditioning on the trait	Residual correlations between items
Local trait Independence (unidimensionality)	Measure one thing at a time (may be subscale as well as total score)	Factor analysis of residuals; comparison of person estimates based on two groups of items
Monotonicity	The probability of a positive response to an item (or in the case of polytomous items, the transition from one response category to the next) should increase with underlying ability, as should the total score	Item fit to the Rasch model such as INFIT or other chi-square–based statistics; item characteristic curves; item threshold pattern
Group invariance	Response to an item should be the same across groups when people are at the same level of the trait. Groups could be demographic, clinical (e.g. diagnostic subgroups), or language/country	Differential item functioning
Reliability and targeting	Reliability is a reflection of the variability in the sample. High values are required if the scale is to be used for individual assessment. Reliability and targeting are closely related. Targeting reflects the coverage of the scale over the persons. While it is argued that targeting is essential, there are occasions when this may not occur in health outcomes, for example, admission after stroke, compared to discharge after stroke. Merging these samples may overcome the targeting problem	Person separation Reliability Cronbach's alpha Person–item distribution map. The Wright map

### 2.1. Homogeneity

At the core of fitting data to the Rasch model is the notion of homogeneity, that same ordering of items and persons, which is tested by item (and person) “misfit”. Note the direction of activity here, that is, the data are tested to see if they meet the model expectations, a template for fundamental measurement. Fundamental measurement in the context of human sciences is what most people understand by measurement, such as weight or height, where units of measurement are of equal size (36–39).

Misfit to the model means that the item or person is somehow not working/responding as the model expects. With the use of activities of daily living (ADL) as an illustration, activities can be ordered in a hierarchy of easy to difficult (40). The model would predict that the more ability, the greater the probability of achieving a more difficult item/task. For example, using a remote control may be seen to be an easier task than washing the back and neck. That is, more people should be able to operate remote controls than wash their back and neck, given the same level of underlying functional ability, as the latter is a harder task (40). However, a subgroup of people may not reflect this relationship, for example, if they have an impairment of their fine motor function. This will be reflected in model fit, where the level of difficulty of items and ability of persons is not constant between those with and without such impairment, and therefore the probabilistic relationship between persons and items does not hold, compromising homogeneity and so contributing to item and person misfit.

There are many different ways to examine item fit, but the INFIT and OUTFIT statistics are widely used for the test of homogeneity, as are other chi-square–based fit statistics. Fit is how closely the item (or person) conforms to the model’s expectations. INFIT is technically an inlier-sensitive or information-weighted fit. This means that it is more sensitive to the pattern or responses to items targeted at the person's ability, whereas OUTFIT is not constrained in this way and may be affected by items whose difficulties are far away from a person's ability. INFIT and OUTFIT statistics are reported as a mean square where the expected value is 1.0. It is the chi-square value divided by the degrees of freedom. A common error when applying the INFIT and OUTFIT statistics is to ignore the critical interval value for the Type I error. Thus, values of the mean square fit statistic such as 0.7–1.3, which are widely used to ascertain fit to the model, do not necessarily retain a 5% Type I error rate (41, 42). For example, applying Smith's formulae for INFIT, the 0.7–1.3 range only applies to 45 cases for an INFIT statistic if a 5% Type I error rate is to be retained (41). The effect is that, for example, 200 cases should have an INFIT MNSQ range of 0.86–1.14 if the 5% rate is to be retained. The more familiar chi-square fit statistic, such as those used in RUMM, is generally interpreted in the usual way, with fit indicated by a non-significant *p* value (i.e. not different from the model’s expectations), often adjusted via a Bonferroni correction. RUMM also has a residual fit statistic, which is analogous to the OUTFIT statistic such that large values are indicative of misfit and low values are indicative of a more dependent response than expected (43). Unfortunately, there are fundamental problems with INFIT and other chi-square–based statistics, which will be considered later. In terms of examining item fit, “evidence of misfit is therefore often only the first step of a long (and sometimes tedious) journey to find out what is really wrong and what to do about it” (44).

The fit of persons is examined in the same way although often much less reported. It is important to note that the misfit of a group of people may be clinically diagnostic, for example, as above where a specific impairment may affect hand function or more generally where cognitive impairment may affect a range of activities for some people, but not others. Some judgment needs to be made about what to do if there is a substantial group of persons with misfit. Any decision made will depend on a mixture of either empirical evidence from fit to the model (e.g. is there a certain item or person subset that seems to be affected?) and/or clinical knowledge about the group.

### 2.2. Local item (response) independence

One important influence upon fit is the requirement of local independence (45, 46). It can be considered as an “umbrella” term to encompass both response (local item) and trait (unidimensionality) dependency (47). Local item “response” dependency (LID) is where two items are correlated after conditioning on the total score—a partial correlation. Items should be independent of one another after taking into account the total score, that is, there are no other associations but their contribution to the trait being measured. Both response and trait dependency are closely linked (48). For example, clusters of items that are response-dependent can generate spurious (bloated-specific) factors and make the item set appear multidimensional (49). On the other hand, applying a unidimensional model when data are multidimensional may generate LID.

It has been a better understanding of the influence of LID that represents one of the major changes in the application of the Rasch model in rehabilitation assessments over recent times (50). A breach of this requirement can influence dimensionality, the monotonicity of item categories in the polytomous model (threshold ordering): the fit of items to the model and deliver an inflated reliability (49). This suggests that current practice should address LID at the outset, whereas previously this has rarely been the case (50). As such, it has been argued that the investigation of LID could be seen to be as important as the examination of multicollinearity in linear regression analysis (51).

It has also been shown that, in general, the threshold for identifying items that fail this requirement, that is, they are locally dependent, is determined by a value of the residual correlation between a pair of items of 0.2 and above, that is, above the overall average residual correlation (52). Scales with a small number of items are likely to have a negative average residual correlation, so local item dependency can be identified with residual correlation values below 0.2. Unfortunately, historically, many levels of residual correlation have been used to identify LID, ranging from 0.3 to 0.7 (53, 54). The effect of this is unknown but, in most cases, will give rise to at least an inflated reliability, if not a serious misinference of model fit.

For existing scales, testlets or “super items” can be created to absorb LID (50, 55–57). These are simply items added together to make larger polytomous items. It is useful to differentiate the two types of combining items by constraining the use of testlets to where an *a priori* item grouping is available, for example, by sub-domains or clear conceptual groupings. On the other hand, “super items” can be used when items are grouped *post hoc*, for example, from the evidence of dependency derived from residual correlations (47). This latter approach may include just a couple of items. It is important to note that utilising this approach does not alter the items or the total score in any way; it is just a mechanism to overcome the local item dependency.

For the development of new scales (particularly where items are derived from qualitative exploration of the domain under consideration), several items may be potentially locally dependent as it is often necessary to ensure that each theme within the domain is covered initially by several items. Once the data is collected, locally dependent items will be flagged up for removal (or perhaps for making parallel forms), contingent on other information such as item fit or differential item functioning (DIF). Parallel forms are where two separate assessments have different sets of items, but overall the assessments are of the same difficulty. Developing a new scale from a large set of candidate items may provide such an opportunity. Also, a locally dependent item is not necessarily a bad item, just a partial replication of another item, referred to as “redundancy” in earlier work (58–60). Some earlier work also identified item redundancy where item difficulties were the same (19). Care needs to be taken in making this sort of judgment where the items are polytomous, for the item “difficulty” is just the average of the thresholds, and these may have quite a different trait coverage, even where “difficulty” is the same.

### 2.3. Local trait independence (unidimensionality)

Unidimensionality is a requirement for summating any set of items (61). Different software have differing approaches to testing unidimensionality. In the RUMM2030 programme, this is undertaken using Smith's *t*-test approach (62). Essentially, this contrasts two sets of items identified as alternative loading by a principal component analysis (PCA) of the residuals to see if the person estimates associated with these sets differ. In WINSTEPS, unidimensionality was historically reported as the percentage of variance explained by the Rasch model, the first residual factor, or the magnitude of the unexplained variance of the first contrast. Unfortunately, simulated data have shown that high values of variance for the Rasch factor or the first residual factor can be reported when multidimensional data are simulated (63). More recent advice emphasises that the magnitude of the unexplained variance of the first contrast should be below 2 for a unidimensional scale (64). DIGRAM, among other investigations, includes a comparison of observed item correlations with those expected by the model under a unidimensional assumption and uses Per Martin-Löf's test of unidimensionality where a non-significant *p* value is supportive of a unidimensional scale (65–67). There is a caveat that for all approaches, there is a requirement for an adequate sample size to have sufficient power to detect multidimensionality.

For some time, a bi-factor solution has been applied to support a total score where the scale has various sub-domains and can be considered multidimensional (68, 69). A bi-factor solution bases the estimates on what can be thought of as the common first factor, where all items load, but also cross-load onto their respective sub-domain. The approach has been widely applied within classical test theory factor-analytic approaches, including supplementary tests to support “essential” unidimensionality, as well as integrated into item response theory models, for example, in Mplus (70, 71).

In RUMM2030, this approach is also applied by deriving the person estimate from that common factor under conditions where *a priori* subscales are present or where patterns of LID support the construction of super items (72). The subscales (testlets) or super items are created using the “subtest” procedure, which simply groups sets of items together. The bi-factor solution is obtained automatically, even where just two items have been grouped into a super item. Here an “explained common variance (ECV)” is reported (the value “A” in the software), whereby a value less than 0.7 is indicative of requiring a multidimensional model, a value above 0.9 a unidimensional model, and the grey area in between, undetermined, requiring further evidence (73). The ECV is the amount of variance retained in that first general factor, although this does not preclude retaining 100% of the variance, or very nearly so. WINSTEPS has a more complicated routine to create testlets and is done *via* excel. See https://www.winsteps.com/winman/testlet.htm.

### 2.4. Monotonicity

Another possible cause of misfit is the lack of monotonicity, that is, the response order of the categories of polytomous items is not associated with an increase in the underlying trait, indicating disordered thresholds. For polytomous items, analysis of threshold ordering has been an important part of scale analysis (8, 74, 75). However, the issue of disordered thresholds has not been without controversy with one approach emphasising its critical nature, and the other not so (76, 77). Nevertheless, while both positions in the argument recognise the diagnostic importance of disordered thresholds, historically practice has largely fractured into two camps, those that do and do not consider rescoring disordered thresholds, that is, grouping categories to improve fit. The more recent understanding of the impact of LID and multidimensionality upon threshold ordering has further complicated the issue (63). LID and/or multidimensionality can possibly generate disordered thresholds, rather than some fundamental aspect of interpretation by those completing the assessment. Furthermore, the use of testlets or super items renders the ordinary interpretation of threshold ordering invalid as, for example, with two items each of five response options, a score of 5 may come from several combinations of each item’s responses. Disordering of a testlet/super item is rather a function of the amount of local dependency absorbed. Individual polytomous items that are retained after dealing with other aspects of fit should be examined for monotonicity and any insights derived as to why disordering may have occurred.

### 2.5. Group invariance (differential item functioning)

Group invariance is also a key requirement for measurement as the scale under consideration should be invariant by relevant contextual factors, for example, gender (78). Originally called “item bias”, it later became known as differential item functioning (DIF) (79, 80). That is, at any value of the trait the response to an item should be the same, for example, for males and females. DIF can be examined by statistical tests, for example, the Mantel–Haenszel test for polytomous variables in WINSTEPS or the ANOVA of residual-based approach in RUMM2030 (81, 82). The whole process of DIF recognition may be further complicated by the presence of “artificial DIF”, whereby one item with strong DIF forces another item to be biased in the opposite direction or where the DIF cancels out at the scale level (83, 84). Unfortunately, in large sample sizes, where a test is reporting DIF, the overlap of item characteristic curves (ICCs) between groups may be visually negligible. This has led to the suggestion that if DIF is detected by whatever means, it should be tested to see if it is “substantive” (82). If DIF is detected, it may be possible to split the item, for example, where one country differs from others. A country-specific item is created for the country deviating from the ICC curves of others, while the remaining countries are grouped together (9). Substantive DIF can then be tested by comparing the person estimates from the unsplit and split solutions. The solutions must be anchored (linked) in some way, using a non-DIF item in the split solution to force the unsplit solution onto the same metric (or vice-versa). If the difference in person estimates between the two solutions is not significant, then no action needs to be taken, and the unsplit solution can be used. If the difference is significant (a paired *t*-test), the magnitude of difference is reported as an effect size. Recently Rouquette and colleagues (85), using simulation, have indicated that a non-negligible effect on *measurement bias occurs with* an effect size >0.015 (if statistically significant) and higher than 0.1 was considered to have a major effect. While there is no current consensus on what value determines the presence of substantive bias, it would appear from the above work that, where the difference between estimates is statistically significant (which may be driven by a large sample size), an effect size of that difference ≥0.1 would indicate substantive bias.

It is also crucial to consider which contextual variables are appropriate for testing for DIF. For example, in health, if the scale under development was a condition-specific quality of life (QoL) measure, then contextual variables should not be on the hypothesised causal pathway (e.g. fatigue, disability), as their effect upon QOL could be mediated, such that the effect of mediation could manifest as DIF (82). Finally, for DIF, the conceptual basis under consideration should determine whether or not DIF should be resolved (by splitting) or not (86). In the current example given below, the measure of functioning in stroke is intended to tap the information caused by specific factors such as side of stroke, aphasia, and dysarthria (all on the causal pathway), and, as such, DIF should not be resolved for these factors.

### 2.6. Reliability

Reliability is a fundamental attribute of any assessment scale. Traditional test reliability is the “true person variance/observed person variance” for the sample on a set of test items (87). Most commonly in traditional test theory reliability is reported as Cronbach's alpha and/or internal consistency (88). WINSTEPS “person separation reliability” indicates how well persons are differentiated on the measured variable. It is based on the same concept as Cronbach's alpha and is based on the variance in the metric rather than the ordinal, unlike alpha (39). WINSTEPS also has a “person separation index” (PSI), which is another way of describing how persons are differentiated on the measured variable. This value can be used to determine the number of discrete strata (either for items or persons) where the number of strata = [(4*separation index) + 1]/3 (89).

RUMM reports a “person separation index (PSI)”, based on the same concept as Cronbach's alpha, but again on the metric (43). RUMM also reports Cronbach's alpha when data are complete, although there is a mechanism to remove cases with missing items to obtain alpha. If the data are normally distributed, then the PSI and *α* will be similar, but otherwise PSI will deviate (usually lower) if the data are skewed. Bland and Altman (90) report that for individual use, the level of person reliability should be 0.9 and above.

Associated with reliability is the concept of targeting. In an educational test, item difficulties will be targeted around the expected student ability level. It would not help to grade student ability if all items were very easy or very hard. In rehabilitation assessment, it would be of little use presenting, for example, ADL items to those starting stroke rehabilitation, where those item difficulties would be consistent with the ability of those being discharged after successful rehabilitation (although this is sometimes done, leading to skewed distributions at either admission or discharge). Properly targeted assessments are crucial, as they can influence reliability. The more off-target, the lower the reliability (91). Conversely, if the patients are more homogenous in their response (they are all at the same level of ability), then reliability can also be reduced.

A visual interpretation of targeting can be found in the Wright map, which displays on the same metric the distribution of persons on the left side and items (and/or thresholds) on the right side (92). The map in RUMM2030 is usually presented as an abridged version, showing a person–item distribution, but without delineating the items, although this is also available. The hierarchical ordering of item difficulty can make an important contribution to construct validity where such ordering is consistent with the theory underlying the scale. Some care needs to be taken when looking at the hierarchical ordering of items where bias may be present, for example, with LID or DIF. There are also challenges with the interpretation of hierarchy for testlets or super items, particularly when these are used with established scales.

## 3. Other relevant aspects

### 3.1. Item banks

There is now widespread application of item banks in health outcome assessment (93–95). An item bank is a collection of many items, which purport to measure the same trait. It has been used in educational settings for a long time, where exam questions are developed each year and gradually an item bank is built up from which new exam questions can be derived, maintaining the comparability of test difficulty over time (96, 97). It is required to meet the selected model requirements just as any other set of items, and some, but not all item banks, are derived using the Rasch model (98–100). Irrespective of how the items are derived, in order to measure a single trait, it is likely that LID exists, often to a considerable extent.

Specialised computer software will administer the set of items in an iterative fashion, depending on the response to the initially presented item, and subsequent items, referred to as computer adaptive testing (CAT). The principal advantage of CAT is that it reduces the burden upon patients by generating an accurate estimate from relatively few targeted items. However, there can be a substantive problem in the presence of LID. Either the item bank will have been created such that there was no LID in the first place, a considerable challenge if many items are required. This is particularly the case as the recent understanding of the magnitude of residual correlations that define LID is much lower than the usually presented. Or, the software will need to be advised about LID items, such that it can avoid presenting items where an associated LID item has already been presented.

There is another advantage of CAT in the context of those instruments developed to measure “individualised” assessments such as The Schedule for the Evaluation of Individual Quality of Life (SEIQoL), the Valued Life Activities Scale, or Goal Attainment Scaling where respondents are allowed to specify those aspects/items relevant to themselves (101–103). The problem with this is that the items so chosen may have considerable variation in difficulty or impact, so rendering comparisons across individuals impossible. However, if a predetermined item set has been derived and offers the basis for choice, the CAT software can simply have a “not relevant to me” option, which then directs it to seek the next suitable item (104). Thus, irrespective of the items chosen, a derived standard metric will allow comparison across individuals.

### 3.2. Test equating

Test equating is where two or more test scores measuring the same construct are linked in some fashion such that scores are comparable. Where test equating is required, Andrich (105) gives an example whereby just the test scores are used as items. The analysis is just the same as in any other Rasch analysis. For example, if three scales are being equated, then the three items (scale total scores) are fitted to the model in the usual way. These scales must have individually satisfied Rasch requirements and should be considered to be measuring the same construct or trait prior to the analysis, although the analysis itself will confirm this through the unidimensionality test (106–108). It is important to note that the thresholds of these scales must be ordered for the purpose of equating.

### 3.3. Sample size

Sample size can be a difficult issue in rehabilitation assessments, often as a result of relatively rare conditions. Linacre published an article that has had considerable influence on the choice of sample size (109). Depending on the targeting of the instrument, sample sizes of >100 to 243 were suggested, although care also needed to be taken with polytomous scales that sufficient numbers were in each category. Most recently, Hagell (110) has reported sample sizes of 250–500 when using the RUMM2030 programme with unconditional fit statistics, as this avoids problems with Type I error rates with larger samples. However, this does not preclude larger samples, where subsamples could be drawn to represent training and validation samples for cross-validation or where conditional fit statistics are used, which can accommodate much larger sample sizes.

### 3.4. Repeated measures

Where data are longitudinal, consideration must also be given to how dependency arising from repeated measures should be accommodated. The strategy applied will, in part, be dependent upon the sample size available. The data will almost certainly be in stacked format, with data from different time points stacked below each other, so that the same person will appear more than once. If the sample is large enough, a calibration sample can be extracted where a given person is only used once, hence avoiding person-related dependency over time (111). The item parameter estimates from this calibration can then be used to anchor the main sample to provide estimates not biased by any person’s dependency on the data. For those with access to SAS, longitudinal Rasch models have been developed, which accommodate person–time dependency (112, 113).

## 4. Requirements for reporting the methods and results of applying the Rasch model

As well as an informative introduction, both the methods and the results of such an analysis need to be reported in such a way that readers can understand what approach has been used: what indicators of model fit have been chosen, with appropriate references to support the magnitude of such indicators, and what steps were undertaken, if any, to resolve problems with the scale. Depending on the journal, some of these aspects may need to be included in supplementary files, but they should be all included to allow readers (and indeed reviewers) to understand exactly what has been done.

Consequently, a good Rasch paper would include:

### 4.1. Methods

4.1.1. For the development of a new scale/item bank, the theory or conceptual model that formed the basis of item choice and the mechanism chosen to generate such items.
4.1.2. For reviewing an existing scale, the original theory or concepts behind the development of the scale, as well as a description of the scale itself (if available).
4.1.3. The sample under consideration for the Rasch analysis and the sample size.
4.1.4. The parameterisation of the model chosen and why.
4.1.5. The software used for the analysis.
4.1.6. How the requirements of the model were to be tested, with appropriate evidence to support choices, for example:
4.1.7. What level of conditional item dependence was chosen?
4.1.8. What strategies were to be implemented should local item dependency be present?
4.1.9. What type and level of item and person fit were chosen? Relationship with sample size.
4.1.10. What strategies were to be implemented if an item or a person misfit is found?
4.1.11. What contextual variables were chosen to investigate differential item functioning (DIF)?
4.1.12. How was DIF to be analysed?
4.1.13. What strategies were to be used if DIF was discovered?
4.1.14. What test of unidimensionality was used?
4.1.15. If longitudinal, what strategy was employed to deal with repeated measures data?

### 4.2. Results

Reporting the results should follow the order of the approach specified in the methods.

4.2.1. The baseline item and person fit should be reported, initially, if possible as an overall test of fit. Depending on the number of items under consideration, a supplementary table of individual item fit could be produced.
4.2.2. A (supplementary) figure (Wright map) giving the item hierarchy would be useful, as this will give some indication (albeit with unknown bias at this stage) if the item set is consistent with an expected theoretical or clinical ordering of items.
4.2.3. It is also important to report the person fit as a small set of persons who strongly misfit may influence item fit. If persons have to be removed, the revised baseline results should be reported, and consideration as to why those persons did not fit the model expectations should be taken up in the discussion.
4.2.4. Also reporting on the threshold ordering (or lack of it) for polytomous items can be done at this stage, although any action to remediate disordering should wait until after local dependency and unidimensionality have been dealt with.
4.2.5. The local independence assumption should be reported next, including the number of items (if any) shown to be dependent, possibly with a description of a pair of such items. The strategy taken to resolve the dependency problems should be reported, with the resulting fit to the model after such adjustments. It should also be noted, as stated above, that threshold ordering for testlets and super items, which are used to remedy local dependency, cannot be interpreted in the same way as for individual items.
4.2.6. At this stage, the unidimensionality test should be reported, even though DIF may also affect dimensionality. Likewise, the unidimensionality test should not be viewed as a “definite” test of unidimensionality but should be considered alongside an integrated quantitative/qualitative interpretation based on an explicit variable (trait) definition (114).
4.2.7. DIF should then be reported including, when present, the results of the test of substantive DIF, and any subsequent splitting for DIF that is deemed necessary. It will not be possible to test for unidimensionality on the full item set if items have to be split, due to the technical problems of calculating residuals when there are structural missing values due to split items. However, this could be undertaken within groups, for example, separately for males and females. It should be understood at this stage that such a result shows a lack of invariance for gender and that the full data set does not fit the model expectations. Nevertheless, depending on the pattern of DIF, it may be possible to link (anchor) both groups through items unaffected by DIF, hence placing both males and females in the same interval scale estimate.
4.2.8. All these reporting requirements may necessitate a continuing iteration of the tests of fit of data to the model. A summary table of the main iterations and associated fit characteristics (including reliability) should go in the main text, while a more detailed fit can be placed in supplementary tables or appendices, depending on the journal and editor preferences.
4.2.9. Word count (and the number of tables and figures) may be more challenging where a new scale is being developed as, for example, the theoretical development of the item set may need to be reported as well as the results of the Rasch analysis. It is sometimes appropriate to report the qualitative results in a separate paper, and then just refer to the main themes in the Rasch paper.
4.2.10. Should data be shown to meet the requirements of the Rasch model, then consideration should be given to producing a transformation table of the raw score to an interval scale latent estimate. It is useful to constrain the range of the latent estimate to the same range as the ordinal scale, albeit showing one decimal point to indicate its interval scale nature.

## 5. A worked example

### 5.1. The setting and patient details

The data for this example represents a secondary analysis of data from the Turkish adaptation of the World Health Organization Disability Assessment Schedule, Version 2.0 (WHODAS2.0) (115). A total of 188 stroke patients were assessed during their stay in the Department of Physical Medicine and Rehabilitation at the Ankara University Medical Faculty, Turkey. Written informed consent was obtained from all patients (or their close relatives), and the study was approved by the Ethical Committee of the Ankara University, Medical Faculty. With a mean age of 63.1 years (12.0), 53.7% were male. On average, they were discharged from rehabilitation after 21.7 days (SD 21.1), ranging from 1 to 82 days, and the disease duration was, on average 35.7 days (SD 31.2), ranging from 3 to 240 days. The greater majority (83.2%) had an ischaemic stroke.

### 5.2. Data preparation and description

The WHODAS 2.0 is based upon the conceptual framework of the International Classification of Functioning, Disability and Health (ICF) (116). It has 36 items measuring six domains of the activities and participation component of the ICF: communication, mobility, self-care, relationships, and life events including work and participation (117). Within the main scale, there are 12 items that form the WHODAS-12 (118). These are detailed in Table 2, both for individual items, the domains they measure, and their location in the overall components of physical and cognitive/social. Given the level of activity limitation following a stroke, 12 items would seem a reasonable response load and a good reason to investigate its psychometric properties in this condition. The only application of data to the Rasch model from the WHODAS-12 so far has been the recent paper involving those with amyotrophic lateral sclerosis/motor neuron disease (119). The analysis in the current data is complicated by structural missing values for the work item where, overall, just 11 people were in employment. As a consequence, only 11 items were analysed, providing a standardised transformed estimate of 0–100.

**Table 2:** WHODAS-12 structure.

Designation	Item	Domain	Component
D1.1	Concentrating on doing something for 10 min	Understanding and communicating	Cognitive/social
D1.4	Learning a new task		
D2.1	Standing for long periods such as 30 min	Getting around	Activities
D2.5	Walking a long distance such as a kilometre or equivalent		
D3.1	Washing your whole body	Self-care	Activities
D3.2	Getting dressed		
D4.1	Dealing with people you do not know	Getting along with people	Cognitive/social
D4.2	Maintaining a friendship		
D5.1	Taking care of your household responsibilities	Life activities	Activities
D5.5	Your day-to-day work/school		
D6.1	How much of a problem did you have joining community activities	Participation in society	Cognitive/social
D6.5	How much have you been emotionally affected by your health condition		

The RUMM2030 Rasch analysis software is utilised, applying the partial credit model (7, 14). Data is read in an ASCII format (essentially text data).

### 5.3. Fit and other considerations

The fit of items (testlets or super items) is determined through a number of indicators, including fit residuals (an acceptable range within ±2.5), *χ*^2^ fit (acceptable probability level >0.05, Bonferroni adjusted), and an ANOVA-based *F*-statistic (acceptable probability level >0.05, Bonferroni adjusted). Misfitting items (domains) are only considered after both LID and DIF have been analysed, and no aggregate strategy can find a solution. Thereafter, independent (i.e. non-LID) items are investigated for threshold disordering to ascertain if this is affecting fit. Response categories are combined if this is found to be the case. DIF for each item and contextual factor is determined by an ANOVA-based analysis, whereas a probability of >0.05 indicates DIF. Should DIF be detected, items can be split, and the difference between split and non-split estimates tested for significance. If significant, the difference is tested for substantive DIF through an effect size >1.0 (85). Consideration is also given to what contextual factors should be included to test for DIF. Grouped age and gender are always important, and education may be important in Turkey as, with this age group, a proportion remains illiterate. The number of days since the stroke (duration) may also be important, and so showing invariance to these factors is essential. Consequently, grouped age, gender, education, and grouped duration are chosen as relevant contextual factors.

Local independence is determined by residual item correlations >0.2 above the average residual correlation (52). Where detected, items can be merged into “super items” or testlets if merging is conceptually based. Unidimensionality is tested through Smith's *t*-test–based analysis (62).

### 5.4. Fit of data to the model

**Figure 2:**
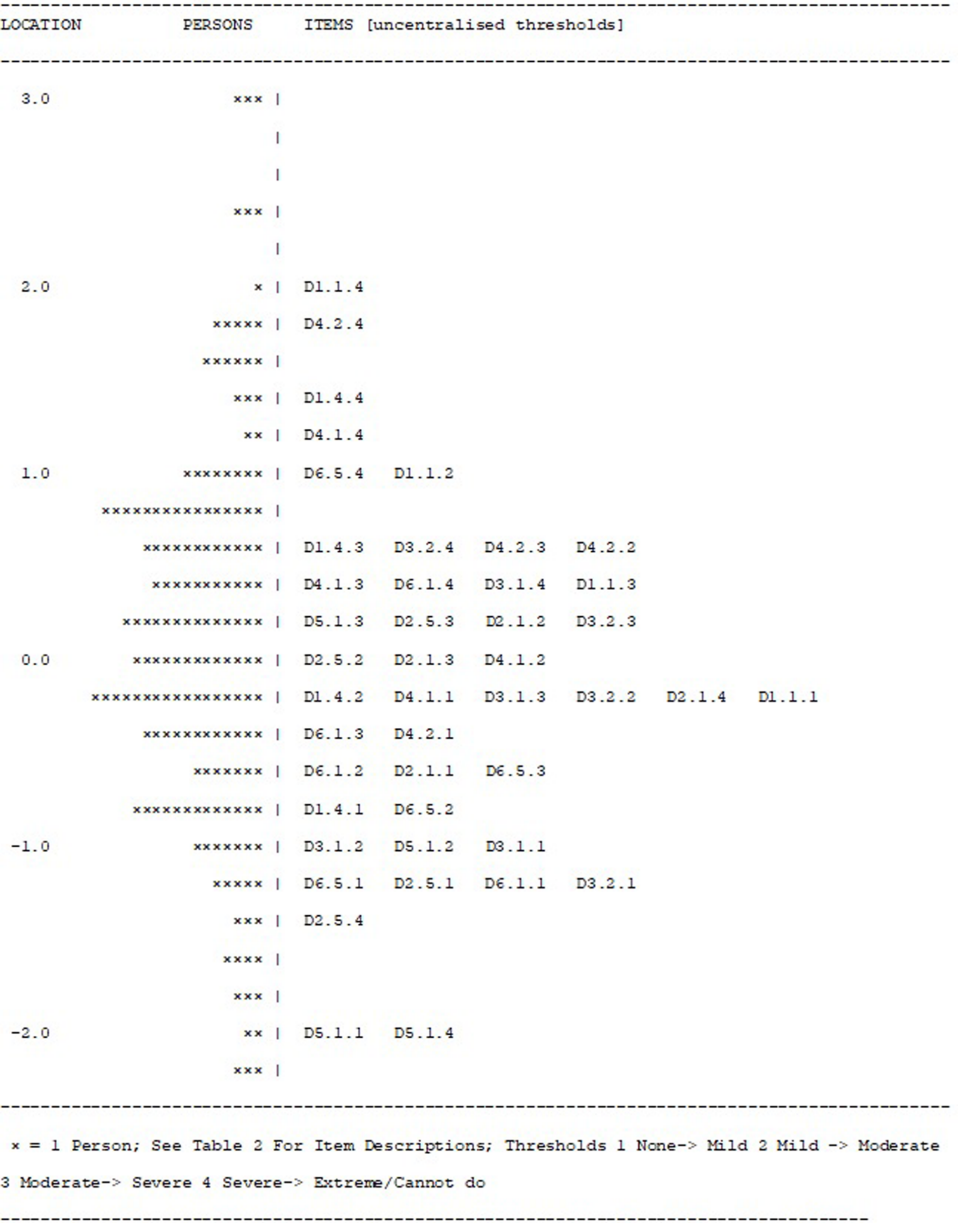
The Wright map.

**Figure 3:**
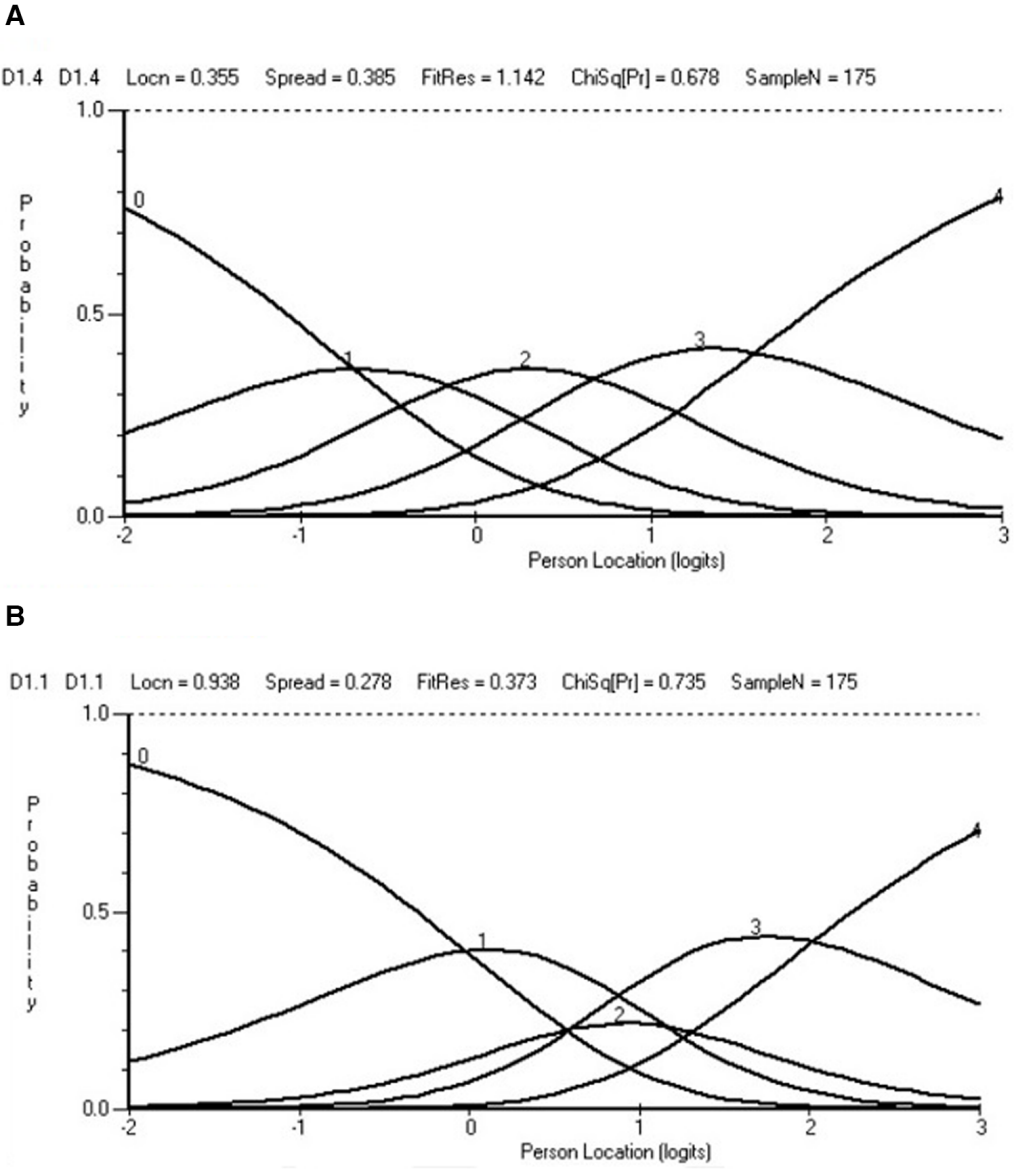
Ordered and disordered thresholds. (**A**) Ordered. (**B**) Disordered.

After reading in the data, the initial fit to the model was examined, at both the person and item levels. A likelihood–ratio test indicated the partial credit parameterisation of the polytomous model to be appropriate. The base analysis is reported in Table 3, Analysis 1, and the individual item fit is reported in Table 4. The Wright map for the 11 items and their thresholds is shown in Figure 2. The item most likely to pick up points on the functioning scale is the transition from none to mild on the item “taking care of household responsibilities” (D5.1). The transition least likely is from severe to extreme on the item “concentrating on doing something for 10 min” (D1.1). None of the items showed a misfit to the model, either by the unconditional chi-square fit or an ANOVA-based fit on the residuals (where fit is indicated by a range of ±2.5). However, six out of 11 items had disordered thresholds. For example, item D1.4 “learning a new task” was properly ordered, with each category having a chance to be the most likely response, whereas with item D1.1 “concentrating on doing something for 10 min”, category 2 was out of order such that the transition (threshold) from category 2 to 3 was at a lower level of disability than that of the transition from 1 to 2, a disordered threshold (Figures 3A,B). The scale was multidimensional, with 14.3% of *t*-tests showing a significant difference. Only four persons showed a misfit to the model through a residual fit statistic > ±2.5.

**Table 3:** Fit of WHODAS-11 to the Rasch model.

	Fit residuals		Chi-square interaction		Reliability		Dimensionality	Local item independence	DIF	ECV
Analysis	Item	Person	χ^2^ (df)	*p*	Person Separation	Cronbach's Alpha	% *t*-tests >5%			
1	1.083	1.014	25.4 (22)	0.273	0.895	0.929	14.3	All pairs within domains	None	–
2	1.178	0.824	20.9 (12)	0.051	0.841	0.882	4.6	d3 + d5	None	0.95
3	0.932	0.930	12.2 (10)	0.275	0.824	0.873	4.0	d2 + d3 +d5 d1 + d4 + d6	None	099
4	0.191	0.706	45.5 (34)	0.090*	0.743	0.820	0.0	–	Education	0.93
Ideal value	<1.4	<1.4		>0.05	>0.7	>0.7	< 5.0	<0.2 below average residual	None	>0.90

* Conditional test of fit.

**Table 4:** Fit of individual items of the WHODAS-11 to the Rasch model.

Item	Location	SE	Residual	Chi-square (df 2)	*p*	*F*-stat (df 2,172)	Probability
D1.1 Concentrating on doing something for 10 min	0.938	0.082	0.373	0.615	0.735	0.110	0.896*
D1.4 Learning a new task	0.355	0.081	1.142	0.776	0.678	0.722	0.487
D2.1 Standing for long periods such as 30 min	−0.062	0.072	−0.607	0.980	0.613	0.656	0.520*
D2.5 Walking a long distance such as a kilometre or equivalent	−0.474	0.073	−1.173	5.069	0.079	3.353	0.037*
D3.1 Washing your whole body	−0.306	0.080	−0.437	2.049	0.359	0.859	0.425*
D3.2 Getting dressed	0.011	0.078	−0.281	0.624	0.732	0.424	0.655
D4.1 Dealing with people you do not know	0.468	0.077	1.256	1.162	0.559	0.409	0.665
D4.2 Maintaining a friendship	0.789	0.082	−0.242	2.887	0.236	2.702	0.129*
D5.1 Taking care of your household responsibilities	−1.071	0.084	−1.090	3.683	0.159	2.056	0.131*
D6.1 How much of a problem did you have joining community activities	−0.358	0.080	−1.374	3.226	0.199	2.915	0.567
D6.5 How much have you been emotionally affected by your health condition	−0.289	0.082	1.893	4.438	0.108	1.874	0.161
Ideal value			<±2.5		<0.05		<0.05

* Disordered threshold.

Considerable local item dependency was found in the data, defined as being 0.2 above the average residual correlation, which was −0.092. These were found for items within each of the domains. For example, D4.1 “dealing with people you do not know” and D4.2 “maintaining a friendship” had a residual correlation of 0.604. Consequently, pairs of items were grouped into their domains, except D5.1 “taking care of your household responsibilities”, which was retained as a single item (as its partner about work in that domain had already been eliminated) (Table 3, Analysis 2). Even so, this item then displayed a significant residual correlation with the self-care domain and was subsequently merged, leaving a five-domain solution (Table 3, Analysis 3). This solution appeared adequate, retaining a reliability (alpha) of 0.87 and 99% of the variance in the data. Unidimensionality was confirmed, and there was no DIF.

**Figure 4:**
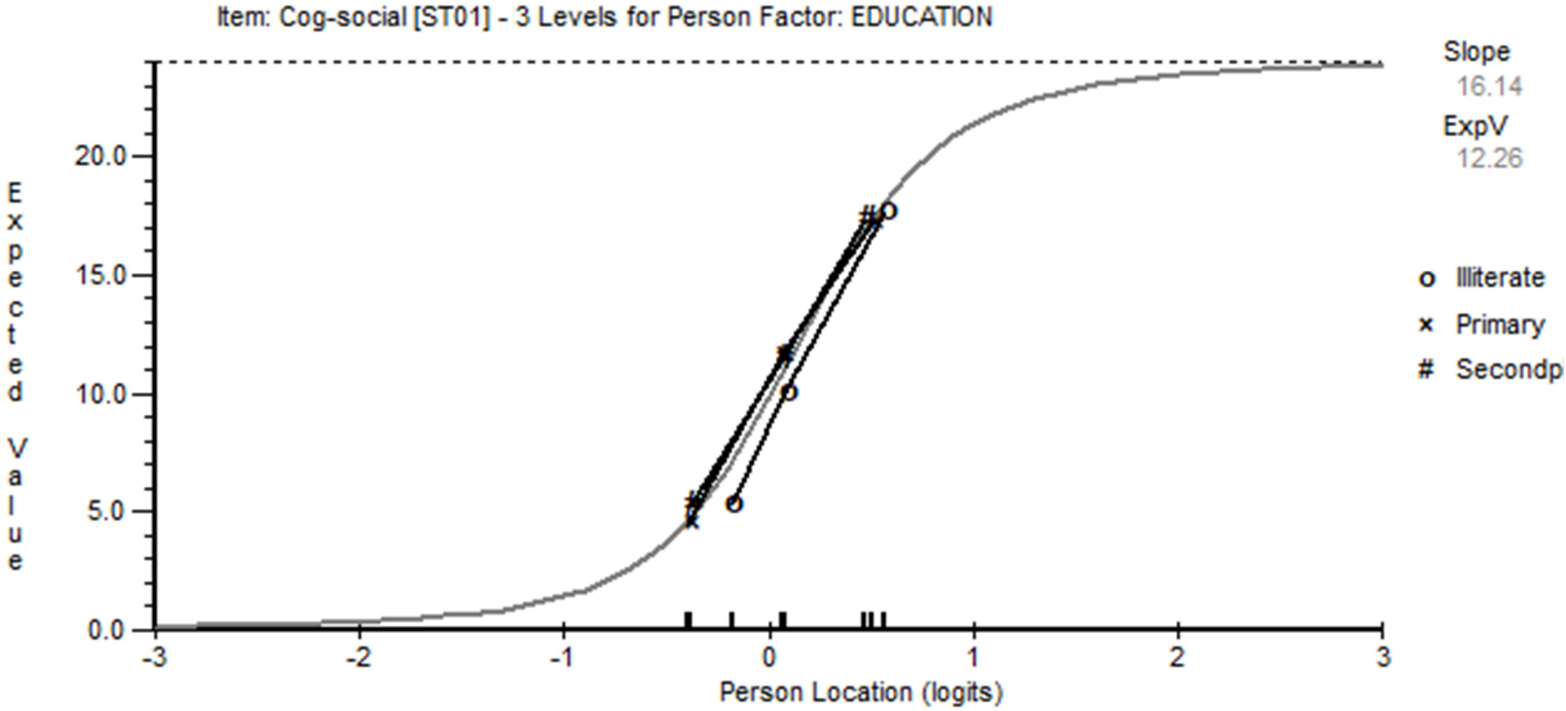
Cognitive/social testlet and educational group lack of invariance.

Nevertheless, local item dependency was observed within the five-domain solution, where the average residual correlation was −0.225, indicating that any positive residual correlation would indicate local item dependency. The dependencies so observed seemed to coalesce into two components, cognitive/social and physical. The analysis was re-run on this basis with the components as testlets and proved adequate with an appropriate conditional *χ*^2^ fit statistic (Table 3, Analysis 4). However, DIF appeared by educational level for the cognitive/social testlet with those who were illiterate, deviating from others (Figure 4). Consequently, the testlet was split on the educational variable, giving a unique variable for those who were illiterate vs. the rest. The unsplit testlet (physical) was used to anchor the unsplit solution to the same metric of the split solution to allow for a comparison of person estimates. This proved significant (paired-test <0.001), and so the effect size of the difference was calculated, showing a value of 0.050 which, given the guidelines above, would not indicate substantive DIF, and so the unsplit solution was retained for purposes of exporting the person estimate in a standardized form of 0–100.

In summary, homogeneity and local independence requirements were met after adjustments to local item independence. Local trait independence (unidimensionality) was supported after dealing with LID. Monotonicity was not supported at the item level but was likely affected by the substantive level of LID grouped within domains. It could no longer be interpreted once testlets were created. No substantive levels of invariance were observed, but reliability was clearly affected by the local item independency, falling from an alpha of 0.93 in the basic item analysis to 0.820 once dependency had been accommodated. The scale was perfectly targeted at the sample as demonstrated by the Wright map (Figure 2).

The limitations of the analysis were primarily associated with the low sample size. Some deviation from model expectations may remain undisclosed due to this. The sample size also precluded cross-validation by splitting the data into training and validation samples. Therefore, the analysis can only be considered a weak confirmation of the internal construct validity of the WHODAS-12 in this stroke sample when 11 items are considered.

## 6. Challenges and future directions

The application of the Rasch model has seen a steady development over time, aided by ever more sophisticated software and a better understanding of how key requirements, such as local item independence affect model fit. As such, “Rasch has laid out a roadmap for building instruments, defining constructs, and making measurements” (120). Increasingly, the term “Rasch measurement theory” (RMT) is applied as the framework for such analysis, originally expiated in Part B of Rasch's book (4, 121). This is an attempt to differentiate it from “item response theory” (IRT), with which it was long associated (122, 123). Andrich (124) has argued that RMT and IRT are two incompatible paradigms. At a simple level, IRT seeks the best model to explain the most variance in the data, adding parameters to the model to facilitate this and, in doing so, allowing item ICCs to cross; in contrast, RMT tests whether or not the data satisfy the requirements of the model, a template for fundamental measurement, requiring non-intersecting item ICCs (19, 125). As such, it would appear advisable to refer to the use of the Rasch model within the framework of Rasch measurement theory (RMT).

There remain a number of issues with the application of RMT, which will need to be addressed in the future. There are problems with the assumptions underlying the distribution of both INFIT/OUTFIT and other *χ*^2^ fit statistics, which cause problems when sample sizes are large. Recent work has shown that Type I error rates for unconditional *χ*^2^ statistics are larger than expected even for moderate sample sizes and are increased for unconditional statistics if *n *≥ 500 (42). Therefore, too many items are regarded as misfitting the Rasch model and may be falsely rejected. Consequently, it has been argued that fit statistics should be based upon conditional inference, which are without bias and less erroneous in large samples (42, 44). Such fit statistics are available in DIGRAM and as a special case in RUMM2030, that is, with two items or two testlets/super items. Nevertheless, all software developers should consider providing conditional fit statistics at the item level.

It is also important to give thought to strategies to deal with item misfit. When developing a new scale (often from a large set of “draft” items), those showing excessive misfit (parameters much worse than other items) should be examined with respect to the theory underlying the development, as well as other indicators such as local item independence and DIF. In fact, decisions about the item should only be taken when all the available indicators are in view, so an informed judgment can be made as to the possible cause of misfit. One recent example of this reported that an overview of all relevant fit parameters was constructed *via* a spreadsheet, a free copy of which was available from one of the authors (126). The hierarchical ordering of items should also be considered at this stage, as this may also give guidance to items most consistent with theory if choices are to be made between items for retention. Some care need to be taken here, as at an early stage of development bias may affect item hierarchy, but if all the information is available, as in the spreadsheet idea above, then this can be taken into account. If the scale under consideration is an existing scale, then item deletion should be the very last option considered, as this will obviously change the instrument and make comparisons with the original difficult. Nevertheless, there are occasions when such action is desirable to avoid inferential bias (127).

While item banks and CAT have appeared over the years, they have been mostly related to their development and validation, sometimes with simulated data (128, 129). There remain substantial challenges to introduce such assessment into routine clinical practice, either within clinics or for follow-up. One example is the use of tablet computers to enable clinic-based assessments (130). The opportunities provided by the convergence of CAT and telemedicine largely remain to be explored, but web-based assessment as well as mobile phone follow-up should provide development opportunities (131, 132). There are barriers to overcome, not least the local technical expertise for implementation, availability of hardware and software, and the associated costs.

There are other developments to enhance the application of the Rasch model. One example explores the different ways in which the transformation may be presented, for example, basing the transformed unit on the standard error of measurement where one unit on the transformed scale represents roughly one standard error (133). The authors provide a spreadsheet that will automatically calculate different transformations given the raw score, location logit, and standard error of the logit. There is also an increasing suite of programmes within the R language (33) although they are not yet integrated into a single analytical framework.

Issues relating to construct theory also remain with regard to the everyday application of RMT. It has been argued that the process should be theory-driven and data-verified. As such “by building instruments based on a solid substantive theory, the Rasch model provides a rigorous structure for collecting and evaluating evidence related to that theory” (120). More recently it has been argued that “In a unidimensional scale, each item in the scale has a specific value. If the theory is unable to explain variation in these values, the instrument is invalid, and the suitability of the construct theory must be questioned” (134). So, it has been stated that Rasch measurement theory needs an attribute theory, which serves to underwrite the inference by validating the quantitative structure of the attribute (135). While this remains a substantive challenge to both new instrument development, and the evaluation of existing instruments, one recent paper showed that it was possible to predict the item difficulty hierarchy of a set of locally independent items from an assessment relating to activities of daily living (ADLs). It was based on the theories underlying occupational therapy (OT) practice as manifest through the knowledge and judgment of the OTs about the intrinsic properties of those ADLs (40). In other words, their judgment about the intrinsic difficulty of ADL items, derived from a number of parameters (including overall physical and cognitive demands) and applied as a construct specification equation utilising a series of linear logistic test models, confirmed the hierarchical structure of items derived from the data (40).

This concept was taken even further by Stenner and colleagues (136) with respect to introducing the notion of causal Rasch models in the context of reading skills. After determining fit and the appropriate attribute application, here, a causal Rasch model involves experimental intervention/manipulation on either reader ability or text complexity or a conjoint intervention on both simultaneously to yield a successful prediction of the resultant observed outcome, which, in their example, is the count correct. In the health sciences, randomised controlled trials (RCTs) may provide one opportunity to investigate how an experimental intervention, which is expected to change some of the variables on a scale (e.g. walking items on a mobility scale), can predict an outcome, given the items in the scale can be deemed to have a differential impact on the outcome. Those outcomes with clinical levels such as depression may also be amenable to this approach.

Other developments are working to have the Rasch model integral to metrology, as applied in the health sciences (37, 137). Metrology includes the notion of “traceability”, which is about preserving the same unit through different uses of the instrument (138). Given the caveat that the Rasch model is about “specific objectivity”, that is, objective within a specific frame of reference, then unit traceability should be confirmed within that frame of reference, such as those with stroke. It is an empirical matter if the traceability extends to other frames of reference. Elsewhere, one study identified the role of a construct specification equation as a recipe for producing “certified reference materials” for calibration of both the task difficulty and a person's ability (139).

This manuscript is just the latest in a series of guidelines, both in books and peer-revied published papers that have been produced to help clinicians and researchers to understand the Rasch model and how to implement it in general, and for specific clinical groupings, including rehabilitation (25, 27, 39, 43, 91, 119 140–145). These publications bring their own perspective to the practice of applying the Rasch model. It is a practice that continues to evolve, and so those using the Rasch model must be kept as up-to-date as possible and think about the existing challenges that remain to be solved. In this way, the best-quality measurement science can inform rehabilitation practice and research. As such, the rehabilitation-based Rasch community can continue to contribute to its development, just as it made a considerable contribution to its early application in health outcome measurement in general and rehabilitation outcomes in particular.

In summary, confirming the construct validity of any rehabilitation summative assessment involves three stages. Initially, starting with a theory, or at least a hypothesis about the construct being measured (based upon clinical experience), data from a summative assessment intended to measure that construct are evaluated to see if all the requirements of the Rasch model are satisfied (internal construct validity). Secondly, the construct under consideration is supported by external evidence from an attribution theory (external construct validity). Finally, under experimental conditions, the understanding of how the construct is measured can enable a successful prediction of the outcome so observed (criterion-related validity).

## Data Availability

The original contributions presented in the study are included in the article, and further inquiries can be directed to the corresponding author.
